# Comprehensive Profiling of Aseer Medicinal Plants: Connections Between Molecular Identity, Chemical Composition, and Antifungal–Antibiofilm Activity Against Oral Yeasts

**DOI:** 10.3390/microorganisms14040795

**Published:** 2026-04-01

**Authors:** Aisha Shathan, Azhar Najjar, Ali Jourk, Samah Noor

**Affiliations:** 1 Department of Biological Sciences, Faculty of Science, King Abdulaziz University, Jeddah 21488, Saudi Arabia; 2 My Health Medical Polyclinic, Jeddah 21431, Saudi Arabia

**Keywords:** oral mycology, non-*albicans Candida*, *Nakaseomyces glabratus*, antifungal resistance, biofilm inhibition, *Abutilon pannosum*, *Foeniculum vulgare*, methyl salicylate, GC–MS, Aseer medicinal plants

## Abstract

Oral fungal infections resulting from non-*albicans Candida* species and new opportunistic yeasts are increasingly linked to antifungal resistance, especially in individuals with periodontal disease. Bioactive compounds may serve as potential alternatives; nevertheless, there is a paucity of research that has comprehensively assessed their antifungal and antibiofilm efficacy against clinically defined oral yeast isolates. This study aimed to (i) describe the variety and antifungal resistance profiles of oral yeasts isolated from women with various periodontal diseases; (ii) assess four ethanolic extracts of Aseer medicinal plants (*Foeniculum vulgare*, *Solanum incanum*, *Forsskaolea tenacissima*, and *Abutilon pannosum*) for their antifungal and antibiofilm properties; and (iii) correlate phytochemical composition determined by GC–MS with biological activity. Oral samples (saliva and subgingival plaque) were collected from 50 female participants with documented periodontal parameters. Fungal isolates were identified using morphological, biochemical (VITEK 2), and molecular (ITS rDNA sequencing) methods. Testing for antifungal susceptibility was performed according to CLSI guidelines. Plant extracts were evaluated for antifungal activity (disk diffusion, MIC, MFC), antibiofilm activity (crystal violet assay and light microscopy), and phytochemical profiling (GC–MS). Fungal growth was detected in 37 of 50 samples (74%), yielding six yeast species: *Nakaseomyces glabratus* (40.5%), *Candida tropicalis* (18.9%), *C. parapsilosis* (13.5%), *Pichia kudriavzevii* (10.8%), *Rhodotorula mucilaginosa* (8.1%), and *Aureobasidium melanogenum* (8.1%). *N. glabratus* demonstrated reduced susceptibility to fluconazole. *A. pannosum* and *F. vulgare* exhibited the strongest in vitro antifungal activity (inhibition zones up to 19.2 mm; MIC 0.19–0.78 mg/mL; MFC 0.39–1.56 mg/mL), significantly greater than *F. tenacissima* (*p* < 0.0001). Sub-MIC concentrations of *A. pannosum* reduced *C. tropicalis* biofilm biomass by 59.6%. GC–MS analysis identified methyl salicylate (20.3–40.2%) and cyclohexanol derivatives (8.0–23.2%) as major constituents. Antifungal activity showed a trend in relation to methyl salicylate content (R^2^ = 0.78). However, because only four plant extracts were included, this relationship should be interpreted as a descriptive observation rather than a statistically testable association. Ethanolic extracts of *Abutilon pannosum* and *Foeniculum vulgare* demonstrated significant in vitro antifungal and antibiofilm activity against clinically relevant oral yeasts, including azole-tolerant *Nakaseomyces glabratus*. The observed trends between phytochemical composition and biological activity warrant further investigation into their potential as adjunct therapeutic agents for oral fungal infections. Further studies are required to confirm these results and see if they can be used in therapeutic settings.

## 1. Introduction

### 1.1. Oral Fungal Infections and Clinical Significance

The oral cavity is a complex and dynamic microbial ecosystem in which fungi—particularly yeasts of the *Candida* genus and other opportunistic species—can live as commensals or pathogens [1,2]. While *Candida albicans* continues to be the most often isolated species, non-albicans yeasts such as *Candida tropicalis* and *Candida parapsilosis*, *Nakaseomyces glabratus* (formerly *Candida glabrata*), and new species such as *Pichia kudriavzevii* and *Rhodotorula mucilaginosa* are becoming more common as causes of oral candidiasis, denture stomatitis, infections associated with periodontitis, and systemic spread in people with weakened immune systems.

Antifungal resistance is increasing, especially to azole drugs like fluconazole, and non-albicans yeasts are becoming more common in oral infections [3,4,5]. The natural reduced azole sensitivity of *N. glabratus* and its remarkable ability to acquire resistance under selection pressure confounds treatment results [6]. More than 1000 times more resistant to antifungal drugs than planktonic cells, oral yeasts may also cling to teeth, dental prostheses, and mucosal surfaces to create structured biofilms [7,8]. At present, there is a dearth of information regarding the antifungal susceptibility profiles and diversity of oral yeasts in Saudi Arabia, particularly among women with periodontal disease [9]. This study intentionally included women to achieve a more uniform sample and reduce any biological diversity between sexes. Moreover, hormonal and physiological variables have been documented to affect oral microbial composition and *Candida* colonization patterns [4,10]. These women may be at risk due to localized inflammation, microbial dysbiosis, and plausible systemic comorbidities [1,4]. A comprehensive characterization of oral yeast populations through molecular, biochemical, and morphological methodologies is necessary to inform evidence-based treatment approaches.

### 1.2. Plant-Derived Antifungals: Rationale and Current Gaps

Bioactive secondary metabolites with anti-inflammatory, antifungal, and anti-timicrobial properties are abundant in medicinal plants [11,12]. In the Aseer region of south-western Saudi Arabia, traditional medicine has long employed indigenous plants, including *Foeniculum vulgare*, *Solanum incanum*, *Forsskaolea tenacissima*, and *Abutilon pannosum*, to address oral infections, inflammation, and wound healing [13,14,15]. Notwithstanding their conventional application, the scientific proof of their antifungal efficacy against clinically significant oral yeasts is still insufficient. Previous investigations have largely focused on *C. albicans*, with limited systematic evaluation of:

Activity against emerging non-albicans and azole-resistant species [16,17];

Antibiofilm effects at sub-inhibitory concentrations [18];

Correlation between phytochemical composition and biological potency (structure–activity relationships) [19];

Integrated testing using standardized CLSI protocols in clinically characterized cohorts [20]. In the Aseer area of southwestern Saudi Arabia, traditional medicine utilizes a range of natural herbs to treat infections and inflammatory diseases [13,14,15]. *Foeniculum vulgare* is well researched for its phytochemical composition and antibacterial activities. Additional therapeutic plants, including *Solanum incanum*, *Forsskaolea tenacissima*, and *Abutilon pannosum*, have been documented in ethnobotanical and phytochemical research examining plants having antibacterial properties [11,13,14]. According to these data, four plant species were chosen for further examination in the current study.

### 1.3. Study Hypothesis and Objectives

We hypothesized that ethanolic extracts of four Aseer medicinal plants, particularly those rich in methyl salicylate, cyclohexanol derivatives, and terpenoids, would exhibit potent antifungal and antibiofilm activity against clinically relevant oral yeasts, including azole-tolerant *N. glabratus*, and that phytochemical composition would correlate with biological activity.

The specific objectives were to:

Isolate and characterize oral yeasts from women with different periodontal conditions using morphological, biochemical (VITEK 2), and molecular (ITS rDNA sequencing) methods;

Determine antifungal susceptibility profiles of representative isolates according to CLSI guidelines;

Evaluate antifungal (disk diffusion, MIC, MFC) and antibiofilm activities of four Aseer plant extracts;

Characterize phytochemical profiles of active extracts using GC–MS and correlate composition with biological activity;

Assess antibiofilm effects using light microscopy.

This study integrates oral mycology, antifungal resistance profiling, and phytochemical–bioactivity correlation in a clinically characterized Saudi cohort.

## 2. Materials and Methods

### 2.1. Study Design and Ethical Approval

This study was cross-sectional and conducted in a laboratory setting, according to the Declaration of Helsinki and received approval from the Institutional Review Board (IRB) of the Faculty of Dentistry, King Abdulaziz University, Jeddah, Saudi Arabia (Protocol Code: 45\97671). Before collecting samples, all subjects gave their written consent.

### 2.2. Participants and Clinical Evaluation

A total of 50 female participants (18–50 years; mean age 27.4 ± 8.7 years) were recruited for this study. All participants underwent clinical periodontal evaluation, including probing pocket depth (PPD), clinical attachment loss (CAL), bleeding on probing (BOP), and plaque index (PI). Participants were categorized according to periodontal status as clinically healthy, dental caries, or mild gingivitis (Stage I) [21].

### 2.3. Sample Collection

Aseptic sampling was performed. Fungal specimens were collected from all 50 participants (*n* = 50) using sterile swabs from the dorsal surface of the tongue and gingival sites, along with whole saliva collection, following standard mycological sampling procedures. The samples were transported on ice and processed within two hours [1,4]. Samples obtained from the tongue, gingival sites, and saliva were processed and cultured separately to allow source-specific isolation and analysis.

### 2.4. Fungal Isolation and Primary Characterization

Aliquots (100–200 μL) were inoculated onto SDA and PDA supplemented with chloramphenicol (50 mg/L) and gentamicin (40 mg/L). The plates were kept at 37 °C and 25 °C for 48 to 120 h. Pure colonies were subcultured and characterized macroscopically (color, texture, elevation, margin) and microscopically (Gram staining, lactophenol cotton blue wet mounts).

### 2.5. Biochemical Identification (VITEK 2 System)

Yeast isolates were identified utilizing YST identity cards with the VITEK 2 Compact system from bioMérieux in Craponne, France, following the manufacturer’s instructions. Molecular confirmation was performed for isolates showing low discrimination or inconclusive identification results.

### 2.6. Molecular Identification (ITS rDNA Sequencing)

The DNeasy Plant Mini Kit (Qiagen, Hilden, Germany) was used to extract genomic DNA. Universal primers ITS1 (5′-TCCGTAGGTGAACCTGCGG-3′) and ITS4 (5′-TCCTCCGCTTATTGATATGC-3′) were used to amplify the ITS region. PCR amplification was conducted in a final reaction volume of 25 μL, including 12.5 μL of 2× PCR Master Mix, 1 μL of each primer (10 μM), 2 μL of genomic DNA template, and nuclease-free water to achieve the total volume.

The amplification was conducted in a thermal cycler with the following parameters: initial denaturation at 95 °C for 5 min, succeeded by 35 cycles of denaturation at 95 °C for 30 s, annealing at 55 °C for 30 s, and extension at 72 °C for 1 min, culminating in a final extension at 72 °C for 7 min. PCR products (~600 bp) were confirmed using agarose gel electrophoresis, then purified, and sequenced in both directions (Macrogen Inc., Seoul, Republic of Korea). The acquired sequences were examined using BLASTn (NCBI, Bethesda, MD, USA; accessed 2023) in comparison to the NCBI GenBank database. Phylogenetic analysis was performed using MEGA X software (version 10), utilizing the Maximum Likelihood technique based on the Tamura–Nei model with 1000 bootstrap repetitions, including *Saccharomyces cerevisiae* as the outgroup.

### 2.7. Antifungal Susceptibility Testing

The disk diffusion assay was performed to determine the antifungal susceptibility of the fungi to antifungal drugs according to CLSI M44-A2. Antifungal susceptibility testing was conducted on representative isolates of the predominant yeast species identified in this study: *N. glabratus* (*n* = 15), *C. tropicalis* (*n* = 7), and *C. parapsilosis* (*n* = 5). These species collectively represented the majority of the isolates and were thus chosen for testing. These isolates were tested against fluconazole (25 μg), voriconazole (1 μg), itraconazole (10 μg), ketoconazole (10 μg), nystatin (100 U), amphotericin B (10 μg), and caspofungin (5 μg). Inhibition zones were measured in millimeters after 24–48 h of incubation at 37 °C. The antifungal susceptibility results were expressed as inhibition zone diameters (mm) without categorical interpretation. Because disk diffusion breakpoints are not available for all species–drug combinations, the results were reported as zone diameters only. CLSI recommendations advise caution in interpreting fluconazole susceptibility in *N. glabratus* when employing disk diffusion techniques, as broth microdilution (CLSI M27) is the reference method for this species [20].

### 2.8. Plant Material and Extraction

In the Aseer area, the aerial parts of *Foeniculum vulgare*, *Solanum incanum*, *Forsskaolea tenacissima*, and *Abutilon pannosum* (March–May 2023) were collected. Species were taxonomically authenticated, and voucher specimens were deposited. Plant materials were shade-dried, powdered, and extracted with 95% ethanol (1:5 *w*/*v*) at room temperature with intermittent shaking. Extracts were filtered and concentrated under reduced pressure at 40 °C, then stored at 4 °C. Stock solutions (100 mg/mL in 10% DMSO) were prepared and filter-sterilized (0.22 μm) [11,13,22].

### 2.9. Antifungal Activity of Plant Extracts

#### 2.9.1. Disk Diffusion Assay

Antifungal activity experiments were conducted utilizing representative isolates of the major yeast species identified in this study: *Nakaseomyces glabratus*, *Candida tropicalis*, and *Candida parapsilosis.* The majority of the detected isolates were these species, which were then chosen for antifungal activity testing.

Fungal solutions (0.5 McFarland; ~1 × 10^6^ CFU/mL) were swabbed on Mueller–Hinton agar supplemented with 2% glucose. 20 μL of extract (100 mg/mL) was applied to sterile 6 mm disks. Additionally, 10% DMSO was used as the negative control and 25 μg of fluconazole per disk was used as the positive control. Plates were incubated at 37 °C for 24 to 48 h, and the size of the inhibition zones was recorded in millimeters [23,24]. However, inhibition zone diameters obtained by disk diffusion may fluctuate depending on physicochemical characteristics of the tested extracts, such as viscosity and volatility. Consequently, the disk diffusion assay should be considered a semi-quantitative screening method for evaluating antifungal activity of plant extracts [19]. All experiments were performed in triplicate.

#### 2.9.2. MIC and MFC Determination

To determine the MIC values, broth microdilution was performed following the general procedures described in CLSI document M27-A3, which remains widely used in antifungal susceptibility testing studies Two-fold serial dilutions (100–0.195 mg/mL) were prepared in RPMI 1640 medium (Sigma-Aldrich, St. Louis, MO, USA) buffered with MOPS (Sigma-Aldrich, St. Louis, MO, USA) and subsequently inoculated with standardized fungal suspensions (1 × 10^3^ CFU/mL). After 48 h of incubation at 37 °C, the MIC for plant extracts was defined as the lowest concentration that inhibited approximately 90% of visible fungal growth. Due to the complexity of plant-derived extracts as mixtures rather than standardized antifungal agents, this endpoint serves as a pragmatic assessment of antifungal efficacy and should be regarded as a non-standard MIC definition [11,19]. To identify the MFC, samples from wells with no visible growth were subcultured on SDA plates. MFC was defined as the lowest concentration that produced no growth after 48 h [18].

### 2.10. Antibiofilm Activity Assay

Fungal suspensions (~1 × 10^6^ CFU/mL) were inoculated into 96-well plates and incubated at 37 °C for 24 h. Non-adherent cells were removed by washing with PBS. Sub-MIC concentrations of extracts corresponding to 0.5 × MIC of each plant extract were then added and incubated for an additional 24 h. Biofilms were stained with 0.1% crystal violet, solubilized in 95% ethanol, and quantified at 595 nm. Percentage inhibition was calculated relative to untreated controls [23,24]. Each experiment was conducted in triplicate.

### 2.11. Light Microscopy

Biofilms formed on glass coverslips were treated with sub-MIC concentrations, fixed with methanol. The samples were stained with crystal violet and examined under a light microscope at 400× magnification [7,8,19].

### 2.12. GC–MS Analysis

Phytochemical profiling was performed using an Agilent 7890B GC system (Santa Clara, CA, USA) coupled to a 5977A mass selective detector. Samples (1 μL) were injected splitlessly onto an HP-5MS capillary column (30 m × 0.25 mm i.d., 0.25 μm film thickness). Helium served as carrier gas (1.0 mL/min). The oven program was set from 50 °C (2 min) to 300 °C at 10 °C/min, holding for 10 min. Ionization was performed in electron impact mode (70 eV). Mass spectra (*m*/*z* 50–550) were compared with NIST 14 and Wiley 9 libraries (similarity index ≥ 90%) [12,14].

### 2.13. Statistical Analysis

The experiments were conducted in three independent biological replicates (*n* = 3). The results are shown as mean ± SD. One-way ANOVA followed by Tukey’s post hoc test was used to evaluate differences between groups. When significant effects were detected, Tukey’s HSD test was used for pairwise comparisons between treatments. A *p* value < 0.05 was considered statistically significant. Descriptive scatter plots were used to explore potential relationships between phytochemical composition and antifungal activity. Statistical analyses were performed using GraphPad Prism v9.0 [18,25,26].

## 3. Results

### 3.1. Recovery and Prevalence of Fungal Isolates

A total of 50 oral samples were examined, and fungal growth was detected in 37 samples, representing 74% of the cases. Statistical analysis showed no significant difference in fungal recovery between saliva and subgingival plaque specimens (McNemar test, *p* > 0.05).

### 3.2. Morphologic and Biochemical Traits of Fungal Isolates

**Figure 1:**
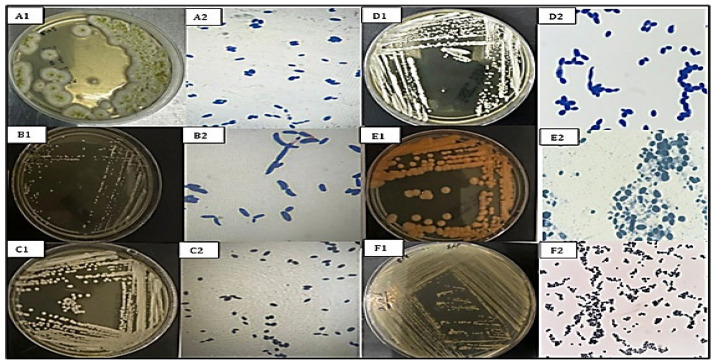
Macroscopic and microscopic characteristics of oral yeast isolates. (**A1**,**A2**) *A. melanogenum*: Dark, smooth, mucoid colonies; oval to elongated cells. (**B1**,**B2**) *C. tropicalis*: Creamy, moist, slightly wrinkled colonies; oval cells with pseudohyphae. (**C1**,**C2**) *P. kudriavzevii*: Whitish, dry colonies; ellipsoidal or cylindrical cells. (**D1**,**D2**) *C. parapsilosis*: Creamy, smooth colonies; small oval cells with short pseudohyphae. (**E1**,**E2**) *R. mucilaginosa*: Orange, mucoid colonies; round to ovoid cells arranged in clusters. (**F1**,**F2**) *N. glabratus*: Creamy, smooth colonies; small round budding cells lacking pseudohyphae.

The morphology of yeast colonies on SDA and PDA showed a variety of differences in color (white, cream, pink, orange), texture (smooth, mucoid, wrinkled), and size variation (2–5 mm diameter at 72 h). Microscopic observation showed oval, ovoid, or cylindrically shaped cells (2.0–8.0 μm). Cells reproduced by budding, and pseudohyphae were observed in some isolates (e.g., *C. tropicalis*). Colony and cellular morphotypes were differentiated into seven morphotypes. VITEK 2 biochemical profiling offered preliminary identification with over 90% confidence for all isolates with consistent profiles that are identical to molecular identification findings. The morphological features of the six species identified are represented in Figure 1, in which the morphology of the colonies and cells on the SDA and PDA media were distinct.

### 3.3. Identification and Phylogenetic Analysis by Molecular Methods

The amplification of the ITS region using PCR generated amplicons of about 600 bp in all the isolates. BLASTn of ITS sequences identified 6 yeast species, 98–100% similar to GenBank reference strains (Table 1). The species distribution was as follows: *Nakaseomyces glabratus* 40.5% (*n* = 15), *Candida tropicalis* 18.9% (*n* = 7), *Candida parapsilosis* 13.5% (*n* = 5), *Pichia kudriavzevii* 10.8% (*n* = 4), *Rhodotorula mucilaginosa* 8.1% (*n* = 3), *Aureobasidium melanogenum* 8.1% (*n* = 3)

**Figure 2:**
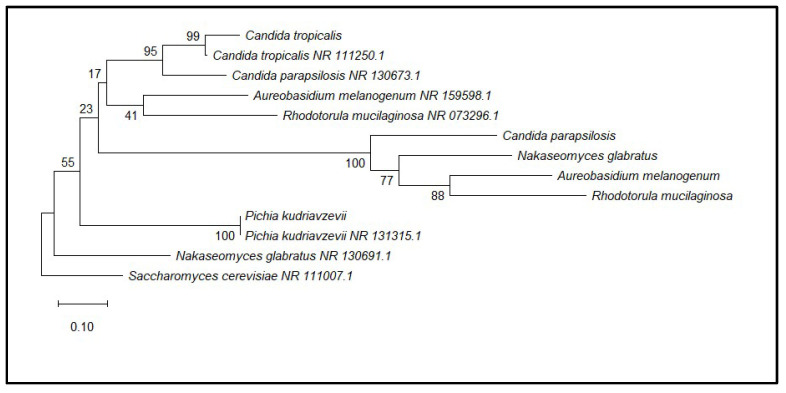
Phylogenetic tree based on ITS rDNA sequences constructed using the Maximum Likelihood method (Tamura–Nei model) with 1000 bootstrap replicates in MEGA12. Bootstrap values (>50%) are shown at branch nodes. Saccharomyces cerevisiae was used as the outgroup.

Phylogenetic analysis divided the isolates into four well-supported groups or clades related to genera *Candida*, *Pichia*, *Rhodotorula*, and *Aureobasidium*, which validated the accuracy of phenotypic and biochemical identifications (Figure 2).

### 3.4. Clinical Isolates and Susceptibility to Antifungals

Disk diffusion testing was conducted in accordance with the CLSI guideline M44-A2. The antifungal susceptibility patterns of the representative isolates (*C. parapsilosis*, *C. tropicalis*, and *N. glabratus*) are summarised in Table 2. The inhibition zone diameters for fluconazole, voriconazole, and itraconazole varied among the tested species. *C. parapsilosis* and *C. tropicalis* showed relatively larger inhibition zones for azole antifungals, whereas *N. glabratus* exhibited smaller inhibition zones for fluconazole (12.1 ± 1.5 mm) compared with the other species. Amphotericin B, nystatin, ketoconazole, and caspofungin generated measurable inhibition zones against all tested isolates.

Because standardized disk diffusion breakpoints are not available for all antifungal agents and species combinations, the results are presented as inhibition zone diameters (mm) without categorical interpretation. The DMSO control disk did not produce any inhibition zones against the tested isolates.

### 3.5. Yields and Phytochemical Profiles of Plant Extracts

The ethanolic extraction yield ranged from 6.4% to 9.8%, with *F. vulgare* showing the highest yield (9.8%), followed by *F. tenacissima* (8.7%), *S. incanum* (7.3%), and *A. pannosum* (6.4%).

GC–MS analysis identified multiple phytochemical constituents, accounting for 96.7–100% of the total chromatographic peak area across the extracts. The major compounds (≥5% peak area) included:

- Methyl salicylate (20.3–40.2%), with the highest percentage observed in *F. tenacissima* (40.2%).
- Cyclohexanol derivatives (8.0–23.2%), most abundant in *S. incanum* (23.2%).

Hexadecanoic acid ethyl ester was detected in selected extracts within the range of 1.8–9.5%.

Methyl salicylate, cyclohexanol derivatives, and terpenoid-related compounds were consistently detected across all four extracts, suggesting a possible contribution of these constituents to the observed antifungal activity (Table 3). The GC–MS chromatograms of the four plant extracts are provided in the Supplementary Materials (Figures S1–S4).

### 3.6. Plant Extracts as Antifungal Agents Against Clinical Yeasts

#### 3.6.1. Antifungal Activity by Disk Diffusion Method

**Figure 3:**
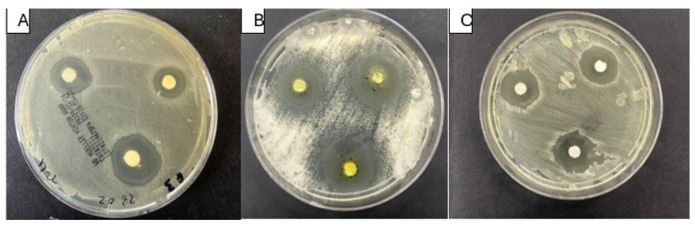
Representative disk diffusion assay plates showing the antifungal activity of *Abutilon pannosum* ethanolic extract against (**A**) *Candida parapsilosis*, (**B**) *Candida tropicalis*, and (**C**) *Nakaseomyces glabratus*.

Of the four extracts, *A. pannosum* and *F. vulgare* showed the most pronounced antifungal activity across all the isolates tested. *F. vulgare* demonstrated one of the highest inhibition zones against *C. parapsilosis* (18.6 ± 0.5 mm), followed by *A. pannosum* (17.1 ± 0.3 mm). *S. incanum* had the highest activity (19.2 ± 0.5 mm) against *C. tropicalis. F. tenacissima* consistently exhibited the lowest activity, and the inhibition zones were between 10.5 and 12.4 mm across the tested species (Table 4 and Figure 3). One-way ANOVA showed statistically significant differences between extracts for all tested species (*p* < 0.0001).

#### 3.6.2. MIC and MFC Results of Antifungal Activity

MIC and MFC were determined using the broth microdilution method according to CLSI document M27-A3. Minimum inhibitory concentrations (MICs) ranged from 0.19 to 3.12 mg/mL. The lowest MIC values were recorded for *A. pannosum* and *F. vulgare* across the tested fungal species. *A. pannosum* exhibited the strongest activity against *N. glabratus*, with an MIC of 0.19 mg/mL and a minimum fungicidal concentration (MFC) of 0.39 mg/mL. *F. vulgare* also demonstrated notable antifungal activity, with MIC values ranging between 0.19 and 0.78 mg/mL. In contrast, *F tenacissima* showed the highest MIC values (1.56–3.12 mg/mL), indicating comparatively lower antifungal potency. As expected, MFC values were generally higher than the corresponding MIC values. Among the tested isolates, *C parapsilosis* and *C.tropicalis* were the most susceptible, whereas *N. glabratus* exhibited comparatively greater resistance (Table 5).

### 3.7. Plant Extracts and Related Antibiofilm Activity

**Figure 4:**
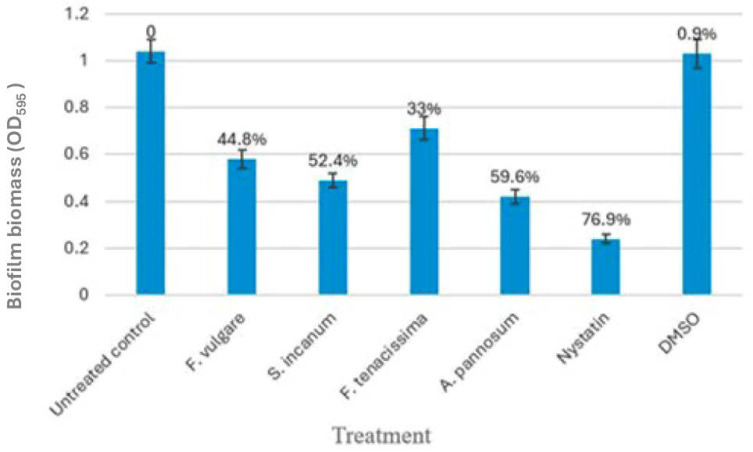
Ethanolic plant extracts antimicrobial activity against *C. tropicalis*. Bar chart of ±SD) of untreated controls and sub-MIC extract-treated groups and controls (nystatin, DMSO). ANOVA 1-way, *p* < 0.0001 vs. untreated control. Error bars represent SD, *n* = 3. Different letters indicate statistically significant differences according to Tukey’s HSD test (*p* < 0.05).

Sub-MIC levels of *A. pannosum* and *S. incanum* decreased the *C. tropicalis* biofilm biomass by 59.6% and 52.4%, respectively, relative to the untreated controls (*p* < 0.0001). *F. vulgare* exhibited 44.8% inhibition, and *F. tenacissima* exhibited the lowest antibiofilm activity (33.0%). Nystatin (positive control) was able to inhibit 76.9%, and 1% DMSO (negative control) did not produce a significant inhibitory effect (Table 6 and Figure 4).

**Figure 5:**
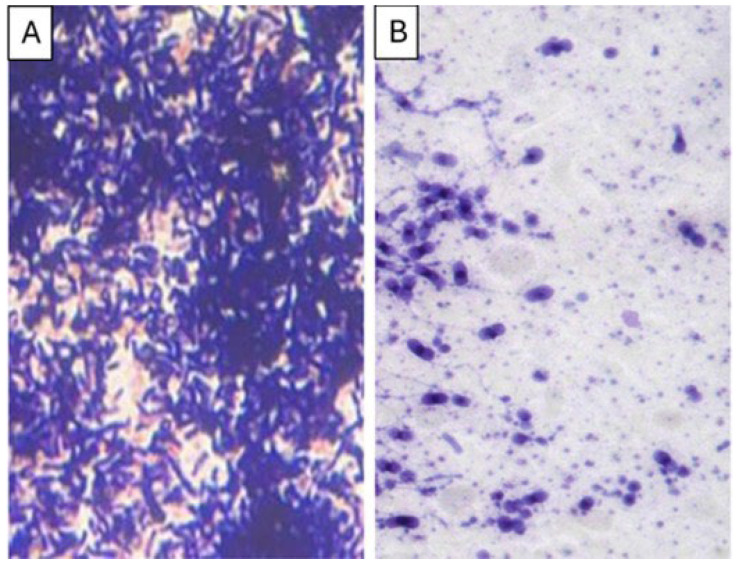
Light micrographs of *Candida tropicalis* biofilms formed on glass coverslips before (**A**) and after (**B**) treatment with *Abutilon pannosum* ethanolic extract at a sub-MIC concentration.

Light microscopy images revealed dense biofilm formation in the untreated control, whereas treatment with the plant extract resulted in a noticeable reduction in cell aggregation and disruption of the biofilm structure (Figure 5).

### 3.8. Correlation Between Phytochemical Composition and Antifungal Activity

**Figure 6:**
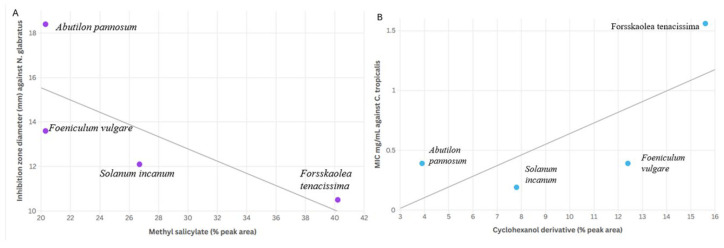
Scatter plots illustrating potential relationships between phytochemical composition and antifungal activity across the four plant extracts. (**A**) Relationship between methyl salicylate content (% peak area) and inhibition zone diameter against *N. glabratus* (R^2^ = 0.78). (**B**) Relationship between cyclohexanol derivative content and MIC values against *C. tropicalis* (R^2^ = 0.71). Due to the small number of extracts analyzed (*n* = 4), these plots are presented for descriptive purposes only.

Potential relationships between phytochemical composition and antifungal activity were explored using descriptive scatter plots. A trend was observed between the relative abundance of methyl salicylate (% peak area) and the inhibition zone diameter against *N. glabratus* (R^2^ = 0.78), as illustrated in Figure 6A. Likewise, a similar trend was observed between the relative abundance of cyclohexanol derivatives and MIC values against *C. tropicalis* (R^2^ = 0.71) (Figure 6B). However, because only four plant extracts were included in this exploratory analysis (*n* = 4), no formal statistical testing was performed, and the observed trends should be interpreted with caution. These findings are therefore presented as descriptive observations only. Further studies, particularly those involving bioassay-guided fractionation and mechanistic investigations, are required to determine the potential contribution of individual phytochemical constituents and their possible interactions.

## 4. Discussion

### 4.1. Oral Yeast Diversity and Clinical Implications

To our knowledge, this study represents one of the few studies on oral yeast diversity in a Saudi female population with established periodontal disorders. The non-albicans yeasts constituted the majority of isolates, particularly *Nakaseomyces glabratus* (40%). These data is consistent with global studies demonstrating shifts in oral mycobiota towards non-albicans species, which may be influenced by antifungal exposure, host immunological condition, and local microenvironmental factors [10,21].

Notably, *Candida albicans* was absent from the tested samples in this investigation. *C. albicans* is frequently identified as the primary oral *Candida* species; nevertheless, several investigations have documented a rising prevalence of non-*albicans Candida* species in oral and mucosal infections [4,17]. Variations in host characteristics, periodontal inflammation, and ecological circumstances within the oral microbiome may affect the distribution of fungal species among populations [21]. Furthermore, research on the oral fungal microbiome has demonstrated that the oral cavity contains a broad array of fungal species, with their relative abundance differing among people and clinical circumstances [10]. The prevalence of organisms like *Nakaseomyces glabratus* and *Candida tropicalis* in this cohort may indicate population-specific microbial trends. However, the lack of *C. albicans* in this dataset must be regarded with caution and requires validation in more extensive research.

The detection of *N. glabratus* in this cohort has important therapeutic implications, as this species is widely recognized for its reduced susceptibility to azole antifungals. In the present study, disk diffusion assays showed smaller inhibition zone diameters for fluconazole in *N. glabratus* compared with the other species tested.

Previous research has linked the intrinsic resistance profile of *N. glabratus* to alterations in ergosterol biosynthesis pathways and the functionality of efflux pumps [27]. The discovery of new species, such as *Pichia kudriavzevii* and *Rhodotorula mucilaginosa*, linked to biofilm formation and opportunistic infections, underscores the necessity for ongoing mycological monitoring [28,29].

### 4.2. Antifungal Activity of Aseer Medicinal Plants

The present findings indicate that *Abutilon pannosum* and *Foeniculum vulgare* exhibited the most potent in vitro antifungal activity among the studied extracts, demonstrating significant activity against azole-tolerant *N. glabratus*. The inhibition zones ranged from 17.1 to 18.6 mm, whereas the minimum inhibitory concentration (MIC) values spanned from 0.19 to 0.78 mg/mL. These results align with those documented for other plant-derived antifungal extracts in the literature [25,30].

The higher effectiveness of *A. pannosum* and *F. vulgare* could be associated with the phytochemicals they contain, such as methyl salicylate, cyclohexanol derivatives, and terpenoid substances. Methyl salicylate has been shown to affect membrane integrity of fungal membranes and pathways for oxidative stress [25,31,32], while chemicals linked to cyclohexanol change how permeable membranes are [33]. However, these interpretations remain speculative, as the present study did not investigate the underlying molecular mechanisms.

Interestingly, *F. tenacissima* had lower antifungal action even though it had a lot of methyl salicylate. These findings suggest that the antifungal effect may depend on the phytochemical matrix as a whole rather than the amount of a single molecule. This supports the idea that plant extracts contain multiple components that may act synergistically.

### 4.3. Antibiofilm Activity

Biofilm development is a key factor in the pathogenicity of oral yeasts, which makes them less susceptible to antifungals [23]. According to the crystal violet assay, low amounts of *A. pannosum* and *S. incanum* reduced *Candida tropicalis* biofilm growth by 59.6% and 52.4%, respectively, in this study. These reductions were statistically significant compared to untreated groups. A limitation of the current work is that the antibiofilm experiment was performed using *C. tropicalis* as a model biofilm-forming strain. Subsequent research should assess the antibiofilm efficacy of these plant extracts against other therapeutically significant species, like *Nakaseomyces glabratus*, recognized for its formidable biofilm-forming capacity. Light microscopy revealed that the extract treatment altered the structure of the biofilm and reduced the number of cells. These findings suggest that there is interference with the structure of the biofilm, but the exact processes that cause antibiofilm action were not directly tested.

Previous research has suggested that chemicals from plants might impact early adhesion processes, the production of extracellular matrix, or the stability of membranes [34,35,36]. It has also been reported that methyl salicylate can modulate the expression of genes in *Candida* species that are linked to biofilm formation [37,38,39]. Even so, more molecular studies need to be done to confirm these processes in the present extracts.

### 4.4. Phytochemistry–Bioactivity Correlation

Exploratory analysis suggested a potential relationship between methyl salicylate content and the inhibition zone diameter against *N. glabratus* (R^2^ = 0.78), whereas a similar trend was observed between cyclohexanol derivative content and MIC values against *C. tropicalis* (R^2^ = 0.71). Even though these relationships are based on only four extracts, they suggest that these components may contribute to the antifungal activity. However, these correlations did not reach statistical significance due to the limited number of plant extracts analyzed (*n* = 4) and should therefore be interpreted as preliminary trends rather than definitive causal relationships.

*F. tenacissima* demonstrated the greatest methyl salicylate concentration (40.2%) however displayed it exhibited comparatively lower antifungal efficacy, suggesting that the biological activity of plant extracts may not be exclusively reliant on the prevalence of a singular component. GC–MS compound identification in this study was based on spectral matching with the NIST mass spectral library, which provides tentative identification according to similarity of fragmentation patterns [40,41,42]. Consequently, compound assignments should be interpreted as putative identifications unless confirmed using authentic standards or complementary analytical techniques. Similar limitations have been widely acknowledged in phytochemical GC–MS studies, where compound identification relies primarily on spectral library comparison rather than definitive structural confirmation [43,44].

Nonetheless, the statistical power of the Pearson test was constrained due to the inclusion of only four plant extracts in the correlation analysis. Consequently, these relationships should be considered preliminary trends rather than statistically significant associations.

These findings support the hypothesis that plant extracts may have bioactivity derived from the interactions or synergistic effects of several phytochemicals working together, rather than just one molecule being the dominant contributor [43,45]. More bioassay-guided fractionation and combination experiments are required to determine what each chemical does alone and how it interacts with the others.

### 4.5. Clinical Relevance

The antifungal and antibiofilm effects seen in vitro suggest that *A. pannosum* and *F. vulgare* extracts should be further investigated as possible adjunctive therapies for oral fungal infections. Notably, they work against *N. glabratus*, which is resistant to azoles.

In any case, these results were only seen in the laboratory. Before clinical application, it has to be tested in vivo to make sure it is safe, bioavailable, pharmacokinetic, and effective.

### 4.6. Limitations and Future Directions

Certain issues should be acknowledged. The sample size was restricted to 50 individuals from a single geographic location, which may limit the generalizability of the results. Secondly, the results are derived from laboratory testing, so they cannot be immediately extrapolated to clinical settings. Third, mechanistic pathways were inferred from the literature rather than validated experimentally.

Future studies should include larger cohorts, bioassay-guided fractionation of active compounds, assessments with various antifungals, and in vivo validation models to evaluate therapeutic efficacy.

## 5. Conclusions

This study provides preliminary information about the types of oral yeast and patterns of antifungal sensitivity in a cohort of Saudi women. The presence of non-*albicans* species, particularly the azole-tolerant *Nakaseomyces glabratus*, emphasizes the need for continued surveillance. In vitro, ethanolic extracts of *Abutilon pannosum* and *Foeniculum vulgare* demonstrated significant antifungal and antibiofilm effectiveness against clinically relevant oral yeasts.

Correlation analysis suggests that methyl salicylate and cyclohexanol derivatives may contribute to this activity. Further experimental and clinical investigations are required to validate these findings and explore potential therapeutic applications.

## Figures and Tables

**Table 1:** Molecular identification of oral yeast isolates and GenBank accession numbers.

Sample No.	Species	Accession No.	GenBank No.	Identity (%)
C37	*Aureobasidium melanogenum*	PX962414	NR159598.1	99.74
C4	*Candida tropicalis*	PX973450	NR111250.1	99.36
C23	*Pichia kudriavzevii*	PX962417	NR131315.1	98.88
C17	*Candida parapsilosis*	PX962415	NR130673.1	99.77
C10	*Nakaseomyces glabratus*	PX962416	NR130691.1	98.24
C11	*Rhodotorula mucilaginosa*	PX962419	NR073296.1	99.24

**Table 2:** Inhibition zone diameters (mm) of antifungal agents against representative oral yeast isolates determined by disk diffusion assay.

Species	FLU	VOR	ITR
*C. parapsilosis*	21.5 ± 1.2	19.6 ± 1.1	18.3 ± 1.0
*C. tropicalis*	20.4 ± 1.3	18.6 ± 1.2	17.4 ± 1.1
*N. glabratus*	12.1 ± 1.5	16.4 ± 1.2	13.7 ± 1.1

Note: FLU = Fluconazole (25 μg), VOR = Voriconazole (1 μg), ITR = Itraconazole (10 μg). Values represent mean inhibition zone diameters ± SD (mm) from three independent experiments. Because disk diffusion breakpoints are not available for all species–drug combinations, results are presented as zone diameters without categorical interpretation.

**Table 3:** Major phytochemical compounds identified in ethanolic plant extracts by GC–MS analysis (peak area ≥ 5%).

Compound	RT (min)	*A. pannosum*	*F. tenacissima*	*S. incanum*	*F. vulgare*
Methyl salicylate	12.68–12.71	20.3	40.2	26.7	20.3
Cyclohexanol, 1-methyl-4-(1-methylethyl)	12.48–12.53	14.7	8.0	23.2	16.3
Hexadecanoic acid, ethyl ester	17.07–17.11	9.5	--	--	1.8
1,4-Benzenediol derivative	15.77	7.9	6.0	3.8	7.6

**Table 4:** Antifungal activity of ethanolic plant extracts against oral yeast isolates by disk diffusion assay.

Fungal Species	*F. vulgare*	*S. incanum*	*F. tenacissima*	*A. pannosum*
	Mean ± SD (mm)	Mean ± SD (mm)	Mean ± SD (mm)	Mean ± SD (mm)
*C. parapsilosis*	18.6 ± 0.5	15.2 ± 0.4	12.4 ± 0.6	17.1 ± 0.3
*C. tropicalis*	14.8 ± 0.6	19.2 ± 0.5	11.3 ± 0.4	16.7 ± 0.5
*N. glabratus*	13.6 ± 0.3	12.1 ± 0.5	10.5 ± 0.4	18.4 ± 0.6

Nystatin (25 μg/disk): 20.8–22.0 mm; DMSO (10%): 0 mm. One-way ANOVA: F = 497.8–612.5, *p* < 0.0001 for all species. Experiments were performed in three independent biological replicates (*n* = 3). Pairwise comparisons were performed using Tukey’s HSD test (*p* < 0.05).

**Table 5:** Minimum Inhibitory Concentration (MIC) and Minimum Fungicidal Concentration (MFC) of Plant Extracts against Fungal Isolates (mg/mL).

Fungal Species	*F. vulgare*		*S. incanum*		*F. tenacissima*		*A. pannosum*	
	MIC	MFC	MIC	MFC	MIC	MFC	MIC	MFC
*C. parapsilosis*	0.19	0.39	0.39	0.78	1.56	3.12	0.19	0.39
*C. tropicalis*	0.39	0.78	0.19	0.39	1.56	3.12	0.39	0.78
*N. glabratus*	0.78	1.56	0.78	1.56	3.12	6.25	0.19	0.39

Values are expressed in mg/mL. Experiments were performed in triplicate (*n* = 3).

**Table 6:** Antibiofilm activity of ethanolic plant extracts against *Candida tropicalis*.

Treatment	Biofilm Biomass $({\mathrm{OD}}_{595}$, Mean ± SD)	% Inhibition
Untreated control	1.04 ± 0.05	—
*F. vulgare* (sub-MIC)	0.58 ± 0.04	44.8
*S. incanum* (sub-MIC)	0.49 ± 0.03	52.4
*F. tenacissima* (sub-MIC)	0.71 ± 0.05	33.0
*A. pannosum* (sub-MIC)	0.42 ± 0.03	59.6
Nystatin (positive control)	0.24 ± 0.02	76.9
DMSO 1% (negative control)	1.03 ± 0.06	0.9

One-way ANOVA: F = 1589.74, *p* < 0.0001. Crystal violet assay, *n* = 3. independent biological replicates. Pairwise comparisons were performed using Tukey’s HSD test (*p* < 0.05).

## Data Availability

The original contributions presented in this study are included in the article/Supplementary Material. Further inquiries can be directed to the corresponding author.

## Supplementary Materials

The following supporting information can be downloaded at: https://www.mdpi.com/article/10.3390/microorganisms14040795/s1, Figure S1: GC-MS chromatogram of the ethanolic extract of *Abutilon pannosum*; Figure S2: GC-MS chromatogram of the ethanolic extract of *Forsskaolea tenacissima*; Figure S3: GC-MS chromatogram of the ethanolic extract of *Solanum incanum*; Figure S4: GC-MS chromatogram of the ethanolic extract of *Foeniculum vulgare*.

## Abbreviations

The following abbreviations are used in this manuscript:

**Abbreviation**	**Full Term**
AMB	Amphotericin B
ANOVA	Analysis of Variance
APC	Article Processing Charge
BOP	Bleeding on Probing
CAL	Clinical Attachment Loss
CAS	Caspofungin
CFU	Colony-Forming Unit
CLSI	Clinical and Laboratory Standards Institute
DMSO	Dimethyl Sulfoxide
FLU	Fluconazole
GC–MS	Gas Chromatography–Mass Spectrometry
IRB	Institutional Review Board
ITS	Internal Transcribed Spacer
ITR	Itraconazole
KET	Ketoconazole
MFC	Minimum Fungicidal Concentration
MIC	Minimum Inhibitory Concentration
OD	Optical Density
PDA	Potato Dextrose Agar
PPD	Probing Pocket Depth
RT	Retention Time
SDA	Sabouraud Dextrose Agar
SD	Standard Deviation
VOR	Voriconazole
YST	Yeast Identification Card (VITEK 2)
