# Supplementary figures and images for: Comprehensive Profiling of Aseer Medicinal Plants: Connections Between Molecular Identity, Chemical Composition, and Antifungal–Antibiofilm Activity Against Oral Yeasts
Source: Microorganisms. 2026 Apr 1;14(4):795. doi: 10.3390/microorganisms14040795 (PMC13119110; doi:10.3390/microorganisms14040795)

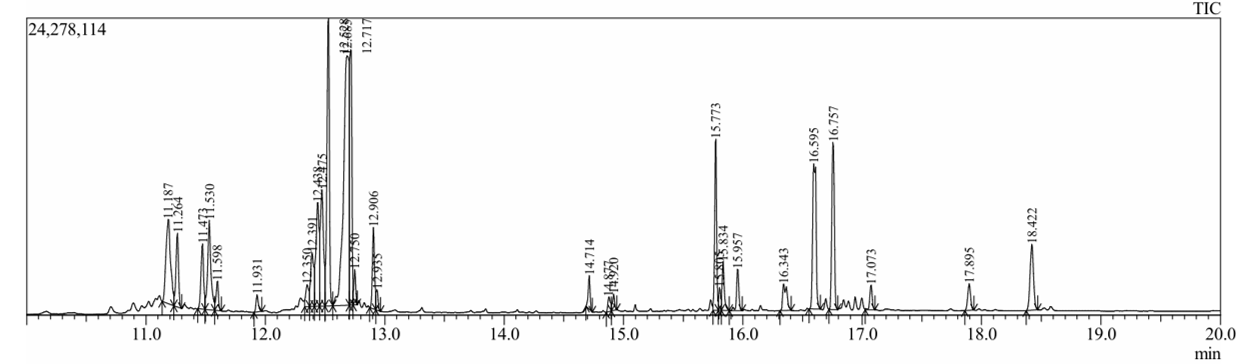

Supplement: Supplementary file 1 [file microorganisms-14-00795-s001.zip › Figure S1. GC-MS chromatogram of the ethanolic extract of Abutilon pannosum.tif]

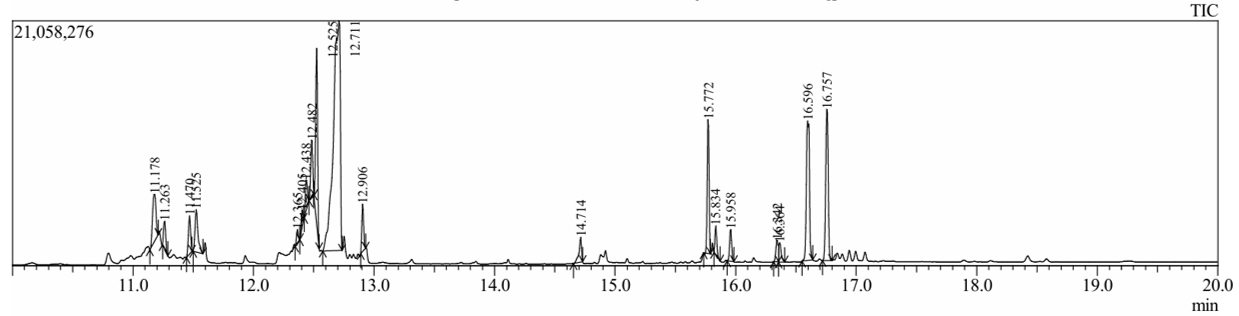

Supplement: Supplementary file 1 [file microorganisms-14-00795-s001.zip › Figure S2. GC-MS chromatogram of the ethanolic extract of Forsskaolea tenacissi.tif]

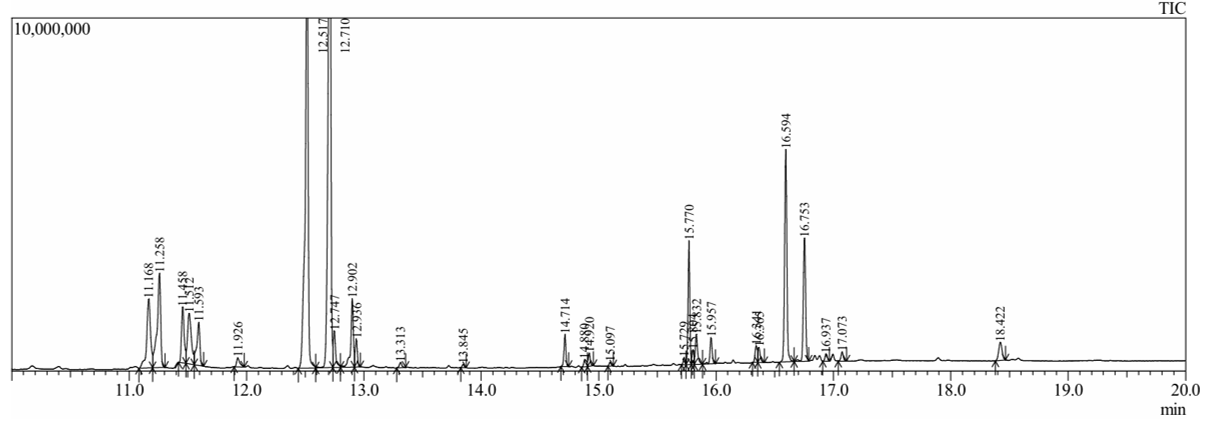

Supplement: Supplementary file 1 [file microorganisms-14-00795-s001.zip › Figure S3. GC-MS chromatogram of the ethanolic extract of Solanum incanum.tif]

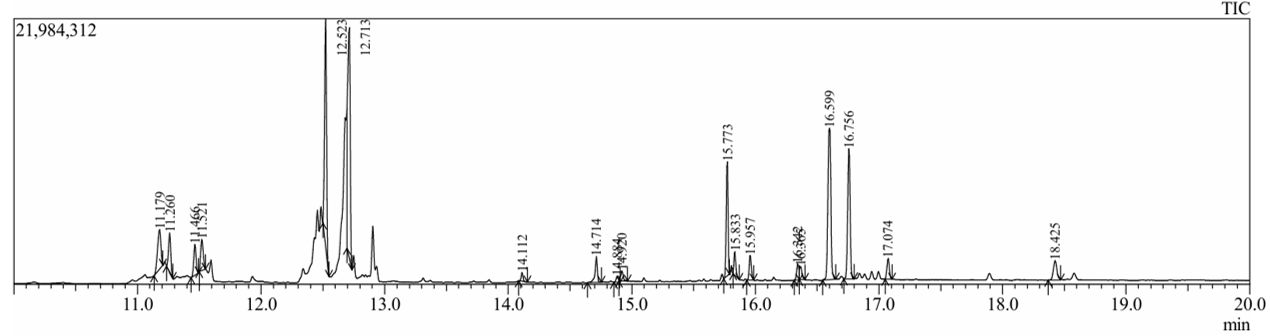

Supplement: Supplementary file 1 [file microorganisms-14-00795-s001.zip › Figure S4. GC-MS chromatogram of the ethanolic extract of Foeniculum vulgare.tif]
